# An Image Encryption Algorithm Utilizing Julia Sets and Hilbert Curves

**DOI:** 10.1371/journal.pone.0084655

**Published:** 2014-01-03

**Authors:** Yuanyuan Sun, Lina Chen, Rudan Xu, Ruiqing Kong

**Affiliations:** 1 College of Computer Science and Technology, Dalian University of Technology, Dalian, China; 2 National Astronomical Observatories, Chinese Academy of Sciences, Beijing, China; Medical University of Graz, Austria

## Abstract

Image encryption is an important and effective technique to protect image security. In this paper, a novel image encryption algorithm combining Julia sets and Hilbert curves is proposed. The algorithm utilizes Julia sets’ parameters to generate a random sequence as the initial keys and gets the final encryption keys by scrambling the initial keys through the Hilbert curve. The final cipher image is obtained by modulo arithmetic and diffuse operation. In this method, it needs only a few parameters for the key generation, which greatly reduces the storage space. Moreover, because of the Julia sets’ properties, such as infiniteness and chaotic characteristics, the keys have high sensitivity even to a tiny perturbation. The experimental results indicate that the algorithm has large key space, good statistical property, high sensitivity for the keys, and effective resistance to the chosen-plaintext attack.

## Introduction

With the increasingly wide reach of the Internet, communications via Internet are getting more frequent. Due to a large number of threats against communications security, information protection has become an important issue. Especially because digital images contain so much information, security for images is a widespread concern. Nowadays, image encryption has been a focus in the research of information security.

Most conventional encryption algorithms put the emphasis on text data or binary data. Therefore, they have highly computational complexity. Because the digital images have special coding structures and large amounts of data, the conventional encryption algorithm may change the original data format in the image encryption. So, among the popular applications of multimedia, research on image encryption has both theoretical and practical significance.

Currently, there are various kinds of image encryption techniques, including image-scrambling-based techniques, data-processing-based techniques, key-based encryption techniques, etc. Some algorithms are based on certain transformation rules. For instance, Shyu used random grids to accomplish the encryption of secret gray-scale and color images [1]. Some algorithms are proposed according to the characteristics of the image itself, such as Yuen’s proposal of a chaos-based joint image compression and encryption algorithm using discrete cosine transformation (DCT) and Secure Hash Algorithm-1 (SHA-1) [2]. Combining the encryption with other data processing technologies, Hermassi introduced a new scheme based on joint compression and encryption using the Huffman code [3]. Among the algorithms utilizing the keys, there has been a great deal of research in chaotic cryptography. For example, Chen produced a key through 3D chaotic cat maps and operated a pixel value with XOR to get the cipher image [4].

In the fractal research field, image encryptions are also explored. Using the fractal set directly as the key is the common method. Kumar proposed a method of encrypting a Mandelbrot set with the RSA method and Elliptical curve [5]. Liu studied a novel fractal cryptographic algorithm based on a fractal model and fractal dimension [6]. Rozouvan encrypted an image with the transformed Mandelbrot set [7]. Lock compressed the original picture for matrix multiplication with the fractal image [8]. Sun used a Mandelbrot set and the Hilbert transformation to generate the random key [9]. Lin encrypted an image by assembling the fractal image additional method and the binary encoding method [10]. Tong proposed an image encryption scheme based on 3D baker with dynamical compound chaotic sequence cipher generator [11].

At the same time, a great deal of analysis has been performed on the image encryption algorithms based on fractal sets or chaos sets. Yuen made a cryptanalysis on secure fractal image coding based on fractal parameter encryption [12]. For some shortcomings in encryption algorithms, Li et al. made the optimal quantitative cryptanalysis of permutation-only multimedia ciphers against plaintext attacks [13] [14].

Many conventional fractal-based encryption methods are combined with fractal coding compression or treat the fractal image as a host image to hide some information, e.g., keys. For the former, fractal coding operation itself may bring the time consumption. This will result in reducing efficiency of the algorithm. For the latter, usually the key length is invariant, which is not flexible and may have some restrictions in the encryption. To meet these challenges, we propose a novel image encryption algorithm. The algorithm uses several parameters to generate the keys with the same size as the plain images and has a good efficiency in the encryption. Firstly, we generate a Julia set and scramble it with the Hilbert curve in bit-level, and then make the scrambled Julia set modulo with the plain image. Finally, the cipher image is obtained by diffusion process. The Julia set is a classical set in fractal theory and can be calculated by several parameters iteratively. For this property, the key is much easier to store and transmit. What’s more, the Julia set has the infiniteness and the chaotic features, so tiny changes of the parameters will lead to dramatic changes of the cipher image. In addition, the diffusion process guarantees that if one pixel value changes, then all the pixels will change, which makes the algorithm resist the chosen plaintext attack effectively.

## The Algorithm

### 1 Julia Set

According to the Escape Time Algorithm, a generalized Julia set can be constructed in the complex plane by the mapping function 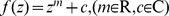. Studies have shown that the Julia set *J(f)* is a closure of the repelling periodic points in the polynomial *f* [15]. The Julia set has sophisticated structures, infinity feature, and self-similarity. When an area of a Julia set border is enlarged, it is still a Julia-like image. What is more, a Julia set has an important feature that *f* is chaotic on the border of Julia set, that is, *f* has sensitive dependence relation to the initial conditions [15]. An arbitrary small perturbation can cause drastic changes in the iterated sequence of *f*. Therefore, we choose the border of the Julia image in the algorithm.

### 2 Hilbert Scrambling

The two-dimensional Hilbert curves are drawn as follows: divide a square into four squares and start the curve from the southwest corner of the center square to the northwest corner; then go to the northeast center, and finally go to the southeast corner. This is one iteration for a Hilbert curve. If we repeat the above process, we can get a curve that fills the whole square. Considering that the Hilbert curve can fill the square and has been proven to be a continuous closed curve, we utilize the curve to scramble the Julia image.

**Figure 1:**
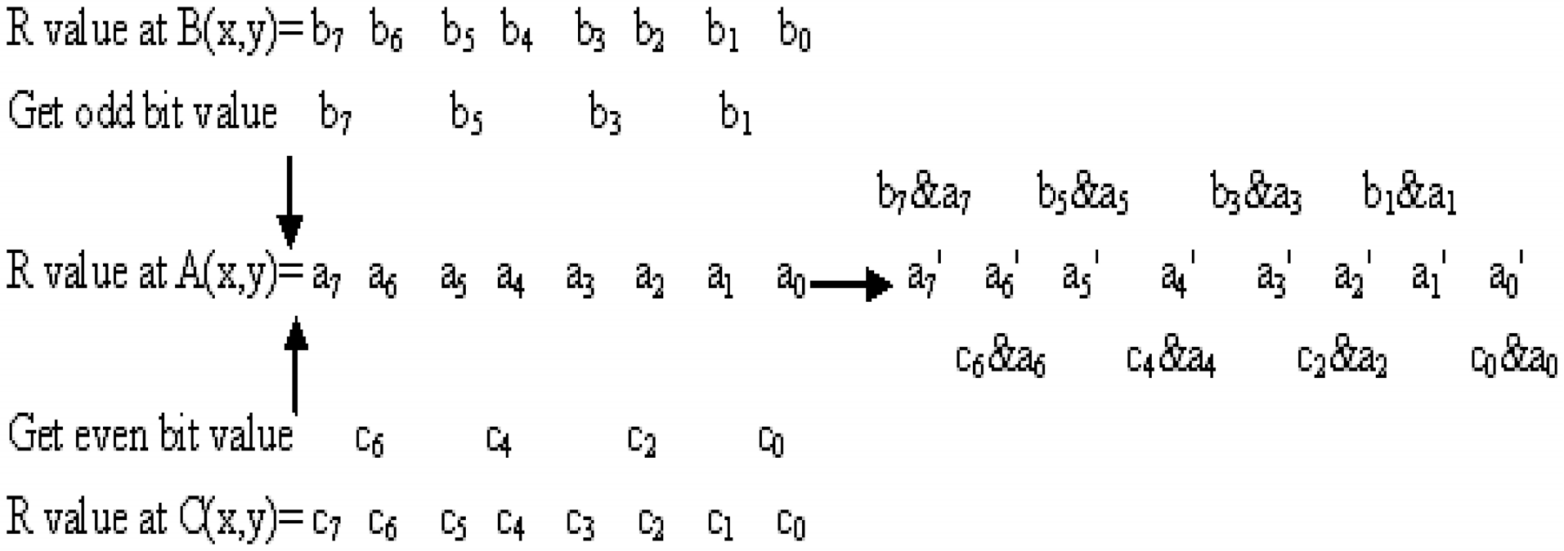
The scrambling process. It is assumed that A, B and C are coordinates in the image, and their pixel values of the R-layer are denoted by (*a_7_ a_6_ a_5_ a_4_ a_3_ a_2_ a_1_ a_0_*), (*b_7_ b_6_ b_5_ b_4_ b_3_ b_2_ b_1_ b_0_*) and (*c_7_ c_6_ c_5_ c_4_ c_3_ c_2_ c_1_ c_0_*) respectively. a^ `^_0_ is obtain from the AND operation between a_0_ and c_0_. Other values are in the same way.



**Figure 2:**
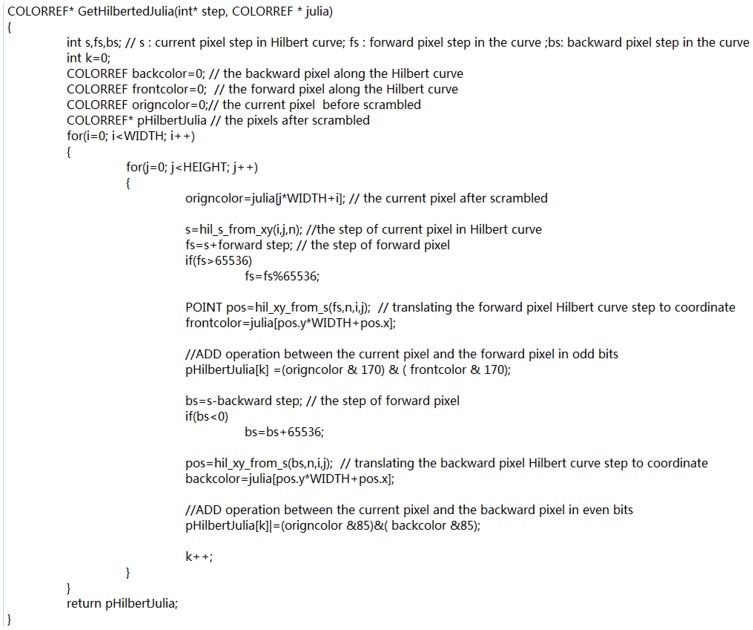
Hilbert scrambling pseudocode.

It is known that the RGB color model is commonly used for representing and displaying the images on the computer screen. The pixel value in each layer can be represented by eight binary bits. Figure 1 displays the scrambling process, where the odd bits are calculated with the forward pixel along the Hilbert curve and the even bits are calculated with the backward pixel along the curve simultaneously. In Figure 1, the values in R layer of pixels A, B, and C are denoted by (*a_7_ a_6_ a_5_ a_4_ a_3_ a_2_ a_1_ a_0_*), (*b_7_ b_6_ b_5_ b_4_ b_3_ b_2_ b_1_ b_0_*) and (*c_7_ c_6_ c_5_ c_4_ c_3_ c_2_ c_1_ c_0_*) respectively. The odd bits of A are calculated through an AND operation with B, and the even bits are obtained in the same way with C, then the pixel value in R layer of A is reset. The scrambling process is recyclable along the Hilbert curve. Equation (1) shows the scramble function.

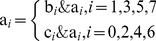
(1)Taking R channel for example, the current pixel value of A is 182, 10110110 in binary. B is the forward pixel along the Hilbert curve with the value 154, 10011010 in binary. C is the backward pixel along the curve with the value 62, 00111110 in binary. Applying Equation (1), we can get the new value of A. Its odd bits are 1×0×0×1× and even bits are ×0×1×1×0. So the final pixel value of A changes to 150 in decimal after scrambling. Figure 2 shows the pseudocode.

### 3 Encryption and Diffusion

The final keys are obtained after the Julia set is scrambled by Hilbert curve. Using Equation (2), we encrypt the plain image with the final keys and get a temporary cipher image.
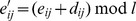
(2)where *e_ij_* is the pixel value of *(i,j )* coordinate in the plain image, *e’_ij_* is the pixel value after encryption, and *d_ij_* is the pixel value in the final keys. Because the image in the experiments is 256-color, the value of *l* is 256.

The diffusion algorithm is also an important image encryption process. Based on a single pixel unit having three layers R, G, and B, we propose a diffusion method. To ensure each pixel in the image can be affected in the diffusion process, the method diffuses the temporary cipher image in horizontal direction firstly, then in vertical direction. Equation (3) shows the diffusion function,
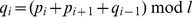
(3)where *q_i_* and *q_i-_*_1_ are the pixel values in the cipher image, *p_i_* and *p_i+_*_1_ are the pixel values in the temporary cipher image. For each layer in the diffusion process, the last pixel value is assigned to the initial value for the next layer iteration, that is 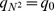
_._ There are no specific values of *q*_0_ in the cipher image and 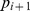 in the temporary cipher image; therefore they are the keys in the diffusion process.

### 4 Decryption

As the proposed algorithm is a symmetric algorithm, the decryption process is the reverse order of the encryption, noting that the iteration order is reversed correspondingly.

The diffusion process starts from the first pixel in the temporary cipher image, with directions from left to right and top to bottom. So, for the inverse diffusion process, it starts from the last pixel in the cipher image, with the directions from bottom to top and right to left. The equation is as follows:
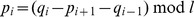
(4)


For the module operation decryption, the order is also reversed and the equation is as follows:
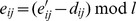
(5)


### The Encryption System

**Figure 3:**
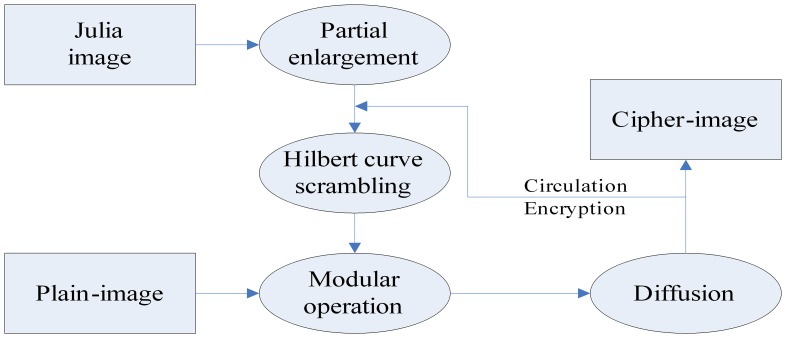
Encryption process. The encryption process can be recycled in the circulation encryption for a better effect. The final encrypted image is the cipher image.

Suppose the initial image is of the size *M×N.* The whole encryption process is as follows:

1. Generate a Julia image by Escape Time Algorithm, select a Julia-like set at the boundary of the Julia set, and then enlarge it to the size of *M×N*;
2. Scramble the Julia-like image by the Hilbert curve to a key image;
3. Encrypt the plain image by the modulo operation with the key image;
4. Diffuse the temporary cipher image;
5. Repeat step (2) - (4) if needed;

An encryption flow diagram of the system is shown in Figure 3.

The details of Escape Time Algorithm are as follows:

1. For complex mapping 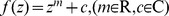, *c* is a complex constant, *L* is the escape radius and *T* is the maximum escape time. *z* is a point in the mapping region of the size 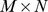. Denote 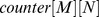 as the two-dimensional array with the initial value 0.
2. For *z*, its coordinate on the screen is 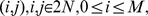 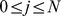.
3. If 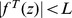, then 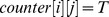 or if 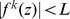, 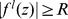, 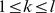, 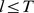, then 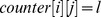.
4. Repeat step (2) and (3) until all points in the mapping region are covered.
5. The color of the point
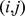
is marked according to 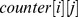.

**Figure 4:**
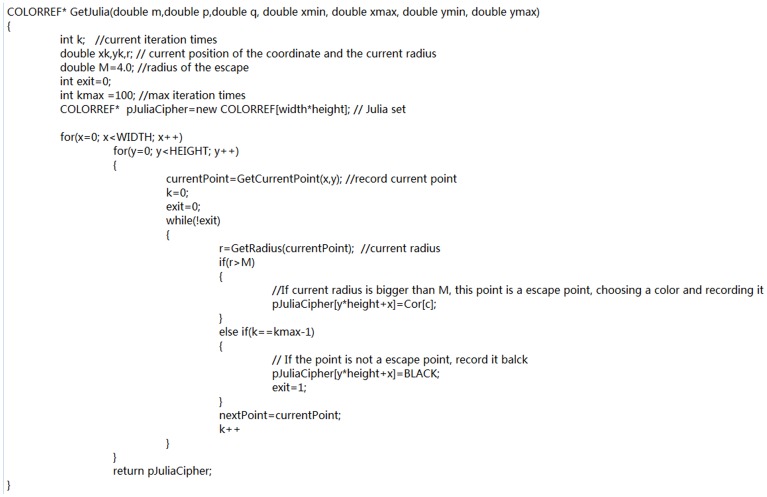
Julia set generation pseudocode.

The pseudocode of the algorithm is shown in Figure 4.

## Experiment Results and Security Analysis

### 1 Simulation Results

**Figure 5:**
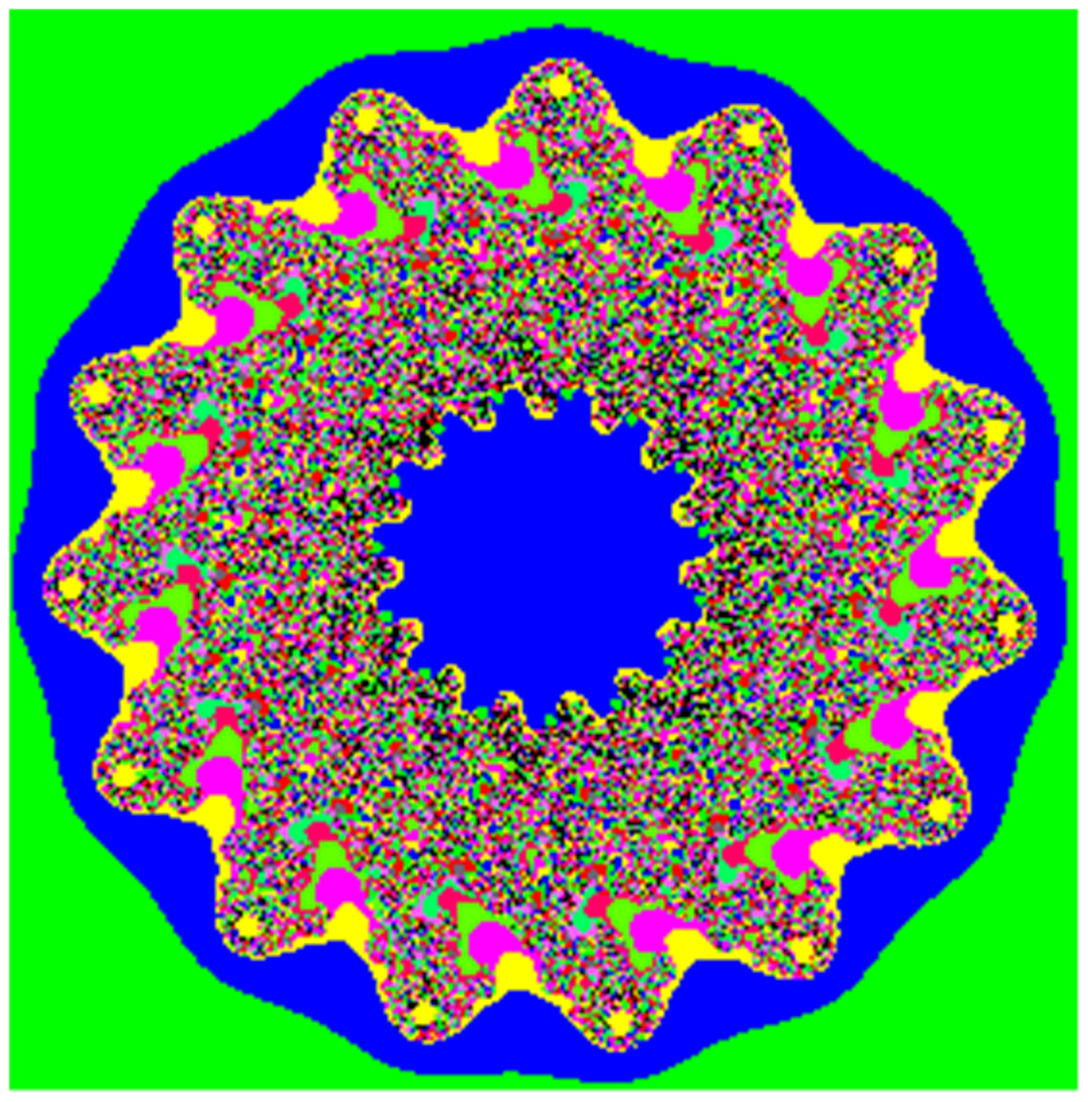
Julia set. The Julia set maps from the complex plane with the ranges from −2 to 2 in X-coordinate and Y-coordinate to the screen with the size of 256×256. The formula is *f(z) = z^m^+c,* in which the *m* = 15, and *c = *0.5–0.7*i*.

**Figure 6:**
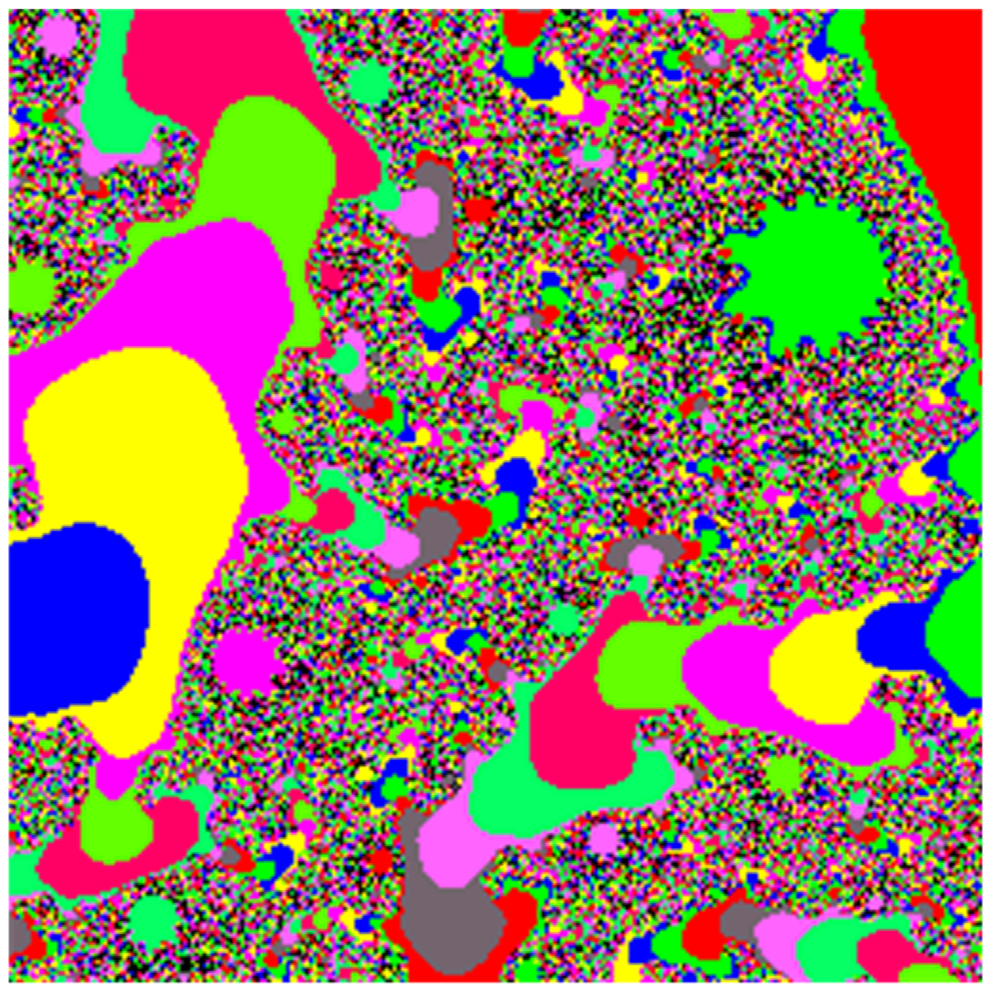
Partial image. This image is partial enlarged one from the Figure 4, and the enlarged area is −0.466866–0.426705 of X-axis, and −0.603235–0.563074 of Y-axis.

We use the Miscellaneous [16] as our database, which consists of 16 color images and 28 monochrome images. All the experiments were conducted on a Core(TM) i5(2.40 GHz) PC. The mapping function of Julia set is *f(z) = z^m^+c* (*m* 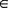 *R*, *c = p+q*×*i,p,q* 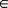 *R*). In our experiments, *m* = 15, *c = *0.5–0.7*i*. The Julia set is shown in Figure 5. The area of −0.466866 to −0.426705 of X-axis and −0.603235 to −0.563074 of Y-axis in Figure 5 is selected to map to a Julia-like set, as shown in Figure 6.

**Figure 7:**
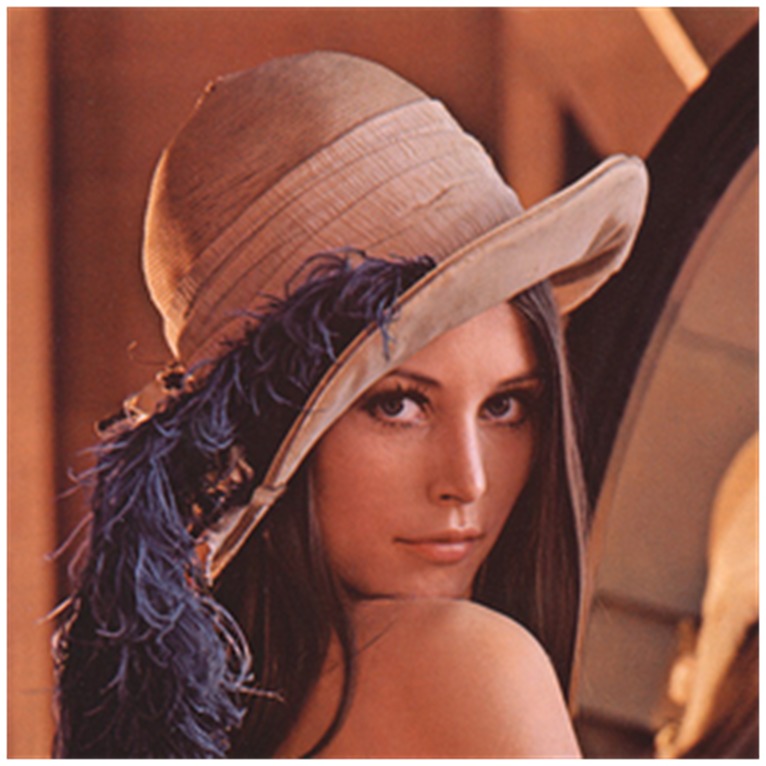
Plain image. The plain image has the same size with the Figure 6.

**Figure 8:**
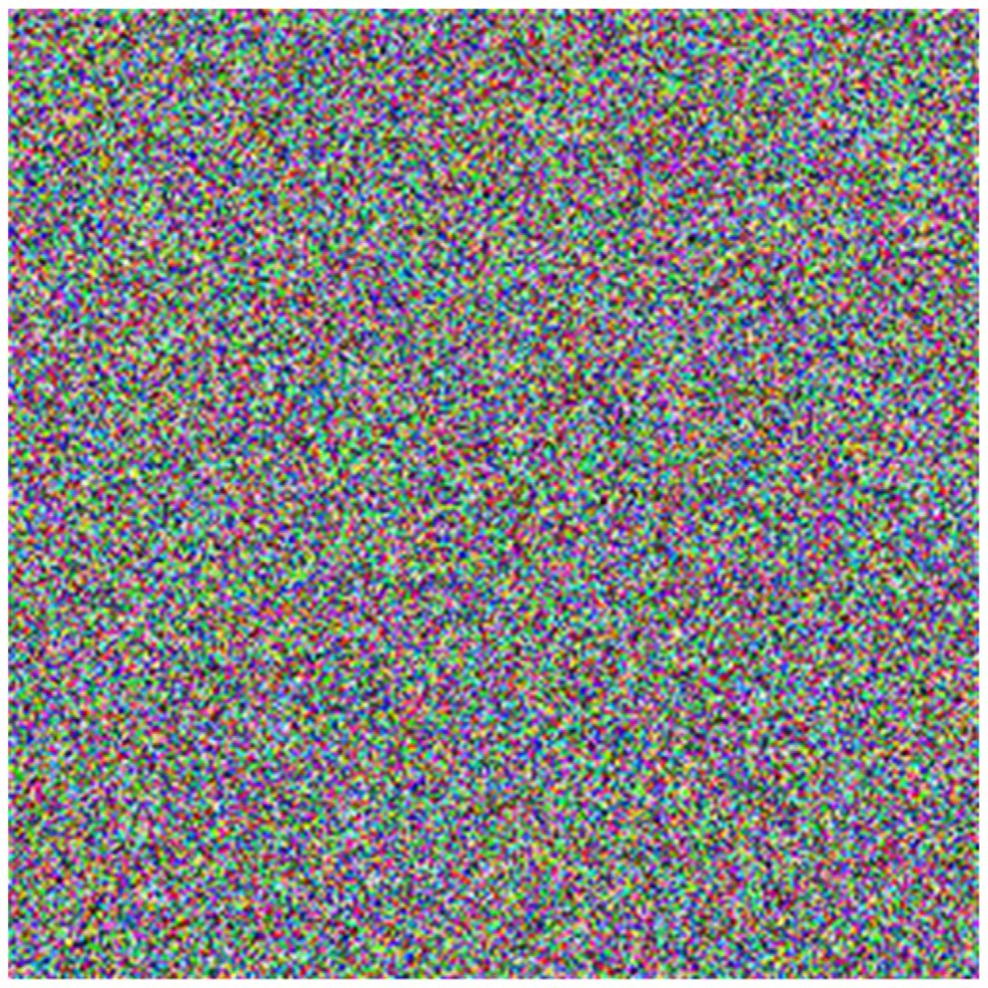
Cipher image. The encryption image is obtained through the scrambling process and the diffusion process.

**Figure 9:**
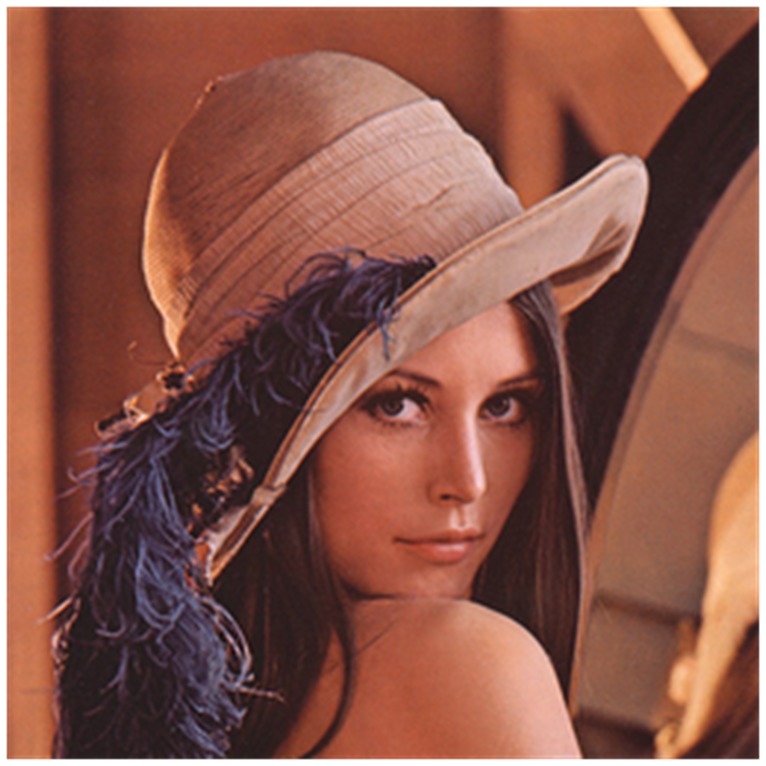
Correct decryption image.

In our experiments, the whole algorithm runs in one iteration. There are two keys in the Hilbert scrambling process, forward step and backward step. They are assigned as 5000 and 9000 respectively. As discussed in Section 3 in the algorithm part, the diffusion process needs only two keys. One is *q*_0_ in R layer of the horizontal direction, and the other is 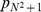 in B layer of the vertical direction. Experimental results show that the images with size of 256×256 cost less than 610 ms for the whole encryption process, in which the Julia set generation costs about 550 ms and the Hilbert scrambling process costs about 15 ms. The experiments produce a satisfying result. In fact, once the Julia set is generated, the scrambling, encryption and diffusion process can be accomplished in a flash. Figure7 shows the plain image, Figure 8 shows the corresponding cipher image and Figure 9 shows the correct decryption result.

### 2 Key Space

The keys in the algorithm consist of the parameters of the Julia set, forward step and backward step along the Hilbert curve, and two diffusion keys. The Julia keys are the mapping parameters *m*, *p,* and *q* (*c = p+qi*) and the area *X_max_*, *X_min_*, *Y_max_*, *Y_min_* (four parameters represent an image area, such as the X-axis scope ranges from *X_min_* to *X_max_*). There are a total of seven keys. They are stored in double data type; the required memory space for one parameter is eight bytes, i.e. 64 bits. The keys for scrambling a Hilbert curve and the keys for diffusion are both in integer data type, with values ranging from 0 to 65535 and 0 to 255 respectively. Therefore, they need 16 bits and 8 bits for storage respectively. As all the above mentioned, the size of the key space is larger than 2^64×7^×2^16×2×k^×2^8×2×k^ = 2^448+48k^, in which *k* denotes the iterations times (k = 1, 2, 3……).

### 3 Key-sensitive Analysis

The whole encryption process includes three sub-processes. They are Julia set generation, Hilbert scrambling, and diffusion process. If the key values change, the corresponding cipher image or decrypted images will be of great difference.

**Figure 10:**
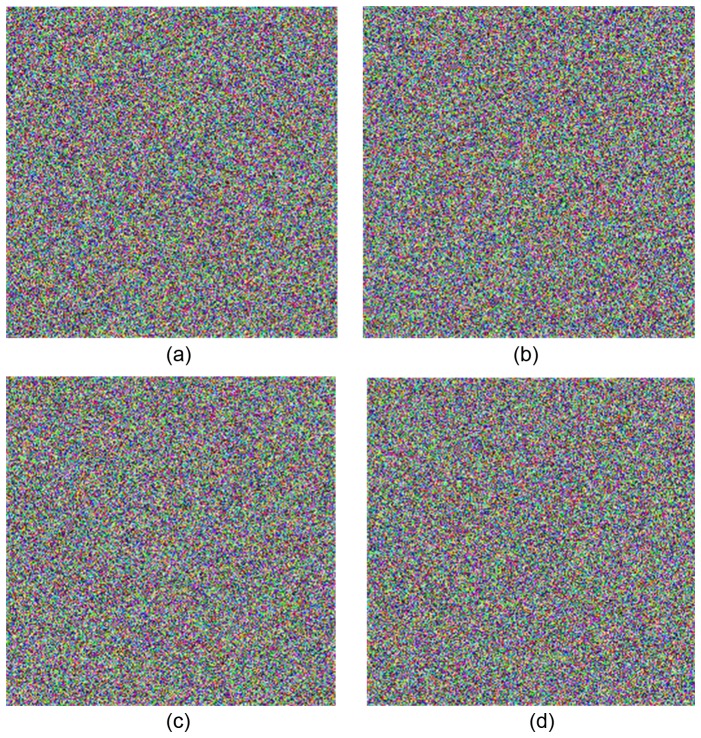
Cipher image with the wrong key and decrypted images. (a) shows the cipher image with the value of *m* changed to 15.000000000000001. (b) is the corresponding decrypted image. (c) is the decrypted image with the value of scrambling key 5001 instead of 5000, which has obvious differences from the Lena image in Figure 9. (d) is the decrypted image with the diffusion key *p_i+_*_1_ changed from 42 to 41 and the value of *q_i_-*_1_ remains 100.

In the decryption, if any key value of the Julia image is changed, the cipher image cannot be decrypted correctly. Taking the key *m* for example, we change the value of *m* from 15 to 15.000000000000001. Figure 10(a) shows the cipher image when *m* value is changed. Comparing the right cipher image (Figure 8) and the wrong cipher image (Figure 10(a)), there are 99.620%, 99.591%, and 99.624% difference in R, G, and B layers, respectively. Figure 10(b) shows the decrypted image by the wrong key. It can be seen that the decrypted image in Figure 10(b) has obvious difference from the plain image in Figure 9, which illustrates the algorithm has a high sensitivity for tiny changes of the initial value *m*.

The scrambling by the Hilbert curve is in bit-level. The keys in this process include a forward scrambling key and a backward scrambling key ranging from 0 to 65535. Figure 10(c) shows the decrypted image with the forward scrambling key 5001 instead of 5000, which has great differences from the Lena image in Figure 9.

The diffusion keys are *q_0_* for R layer in horizontal direction diffusion and 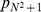 for B layer in vertical direction diffusion. In the experiment, the value of 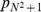 is changed from 42 to 41 and the value of *q_0_* remains 100. Decrypting the cipher image in Figure 8, we get the wrong decrypted image shown in Figure10 (d).

Table 1 shows the different ratio between two decrypted images in R, G, and B layers, respectively. The correct cipher image and the wrong cipher image have great differences when a key value is changed slightly, that means the correct decryption will happen only when all keys are correct. So it is easy to conclude that the keys have a high-sensitivity.

**Table 1:** Different ratio between Lena image and encrypting Lena image with a certain key changed.

	Rate of change in R-Layer	Rate of changes in G-Layer	Rate of changes in B-Layer
key value–m(15 changed to 15.000000000000001)	99.612%	99.612%	99.632%
key value–forward scrambling key(5000 changed to 5001)	99.167%	99.249%	99.196%
key value–qi-1 of R-Layer(80 changed to 81)	99.608%	99.619%	99.622%

### 4 Plain image Sensitivity Analysis

Generally speaking, a chosen-plaintext attack is an attack model in which the attacker obtains the right to use the encryption system, makes a minor change of the plaintext and examines the changes of the ciphertext. The purpose of the attack is to gain some further information to reduce the security of the encryption scheme. In the worst case, the attack could reveal the scheme’s secret keys. If a minor change in the plaintext could cause large changes in the ciphertext, then the aggressive behaviors may be meaningless.

The common standards to test plain image sensitivity are NPCR (the number of pixels change rate) and UACI (unified average changing intensity) [17]. Usually, the plaintext sensitivity will be better if the NPCR value is larger. The formulas are shown in Equation (6) and Equation (7).
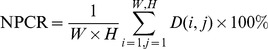
(6)

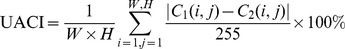
(7)where *C*_1_ and *C*_2_ are the encrypted images, and their corresponding plaintexts have only a one-bit difference in the same pixel before encrypted. The *C*_1_(*i,j*) and *C*_2_(*i,j*) are the pixel value at grid (*i,j*) in C_1_ and C_2_, respectively. And *W* and *H* are the width and height of the images. If *C*_1_(*i,j*) =  = *C*_2_(*i,j*) then *D*(*i,j*) = 1; otherwise, *D*(*i,j*) = 0. Therefore NPCR is to measure the percentage of the different pixels between two images. And the UACI is to test the average intensity of differences.

Table 2 and Table 3 show the plain image sensitivity. We calculate NPCR and UACI values for each pixel LSB (Least Significant Bit) changed in the R channel of the Lena image, Baboon image and Pepper image. Their average NPCR are all about 99.6% and their average UACI are 33.4877%, 33.4175%, and 33.4743%, respectively. Some NPCR and UACI are listed in Table 2 and Table 3.

**Table 2:** The NPCR values for encrypting Lena image.

	Change pixel-value in plain image	NPCR in R-Layer	NPCR in G-Layer	NPCR in B-Layer
(195,112,76) in (0,0)	(196,112,76)	100%	100%	100%
	(195,111,76)	100%	100%	100%
	(195,112,75)	100%	100%	100%
(186,139,124) in (100,150)	(185,139,124)	99.451%	100%	100%
	(185,138,124)	99.976%	100%	100%
	(186,139,125)	100%	100%	99.451%
(69,39,37) in (255,255)	(68,39,37)	100%	100%	100%
	(69,40,37)	100%	100%	100%
	(69,39,36)	100%	100%	100%

**Table 3:** The UACI values for encrypting Lena image.

	Change pixel-value in plain image	UACI in R-Layer	UACI in G-Layer	UACI in B-Layer
(195,112,76) in (0,0)	(196,112,76)	33.46%	33.45%	33.52%
	(195,111,76)	32.16%	33.5%	33.33%
	(195,112,75)	33.45%	32.45%	33.58%
(186,139,124) in (100,150)	(185,139,124)	33.58%	33.71%	33.41%
	(185,138,124)	33.45%	33.28%	33.5%
	(186,139,125)	33.47%	33.56%	33.60%
(69,39,37) in (255,255)	(68,39,37)	33.41%	33.32%	33.46%
	(69,40,37)	33.6%	32.8%	33.5%
	(69,39,36)	33.4%	33.6%	32.8%

The experimental results show that the sensitivity of the plain image is significant. When any pixel bit is changed in one layer, it can influence almost all the pixel values of the cipher image. In this case, the cipher image cannot be decrypted correctly. It is noted that such experimental effects partially owes to the diffusion process in the algorithm. From the above experimental results, we can draw the conclusion that the encryption algorithm can resist chosen plaintext attacks effectively.

### 5 Information Entropy Analysis

It is widely known that the entropy *H(g)* of a message source *g* can be calculated in Equation (8) [17].
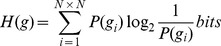
(8)


Where 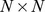 is the amount of the information, *P(g_i_)* is the occurrence probability of the *g_i_* value in all of values. The logarithmic function is to represent the entropy in bit form. If the source sends 2^8^ symbols (containing *g_i_*) with equal probability, i.e., *G* = {*g*_1_, *g*_2_, *g*_3_, ……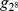}, then the entropy value should be equal to 8. In this case, it is a truly random source. So, the entropy value of an encrypted image should be up to 8.

Table 4 lists the entropy value of the cipher image, which is close to the ideal standard value. It is a clear proof that the encryption system has a good randomness, which indicates it can resist the entropy attack.

**Table 4:** The entropy of the ciphertext.

Cipher image	Entropy in R-layer	Entropy in G-layer	Entropy in B-layer	Average entropy
Lena	7.99728	7.99746	7.99716	7.99730
Baboon	7.99693	7.99695	7.99706	7.99698
pepper	7.99604	7.99457	7.99610	7.99567

### 6 Statistical Analysis

**Figure 11:**
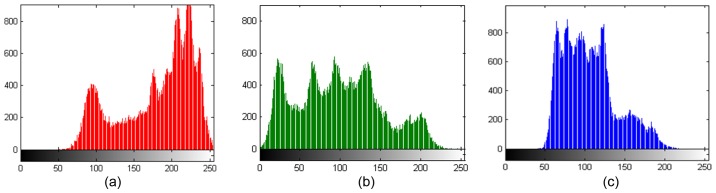
(a), (b), (c) are the R, G, B channel distributions of Lena image, respectively.

**Figure 12:**
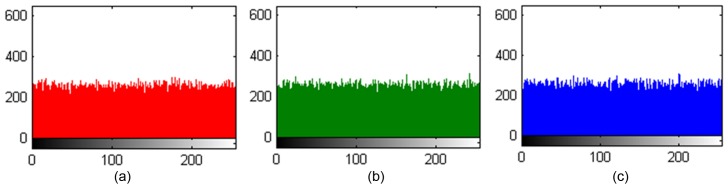
(a), (b), (c) are the R, G, B channel distributions of encrypting Lena image, respectively.

The plain image histograms are shown in Figure 11(a) to Figure 11(c) and cipher image histograms are shown in Figure 12(a) to Figure 12(c), in which the X-ordinate represents the gray-level value and the Y-ordinate represents the occurrence frequency for each gray-level value. The experimental results indicate that each layer’s gray value distribution of the cipher image tends toward equilibrium. These figures demonstrate a uniform distribution of pixel color values for the three image channels, which proves the success of the algorithm in randomizing the output.

### 7 Randomness Test

Table 5 shows that our encrypting Lena image passes the sp800–22 test suite. It proves once again that the cipher image has a good randomness.

**Table 5:** The encrypting Lena image result of sp800–22 test suit for encrypting Lena image.

Statistical test	P-value	Result
Frequency	0.936447	SUCCESS
Block Frequency (*m = 128*)	0.584900	SUCCESS
Cusum-Forward	0.372175	SUCCESS
Cusum-Reverse	0.427874	SUCCESS
Runs	0.602041	SUCCESS
Long Runs of Ones	0.731317	SUCCESS
Rank	0.030824	SUCCESS
Spectral DFT	0.712291	SUCCESS
NonOverlapping Templates (*m = 9,B = 000000001*)	0.292611	SUCCESS
Overlapping Templates (*m = 9*)	0.919983	SUCCESS
Universal	0.819843	SUCCESS
Approximate Entropy (*m = 10*)	0.946813	SUCCESS
Random Excursions (*x = +1*)	0.882358	SUCCESS
Random Excursions Variant (*x = −1*)	0.538752	SUCCESS
Linear Complexity (*M = 500*)	0.278069	SUCCESS
Serial	0.960519	SUCCESS
	0.934595	SUCCESS

## Conclusions

In this study, we have proposed an encryption algorithm combining the classical Julia set and the Hilbert curve. In the algorithm, the Julia set is scrambled in bit-level by the Hilbert curve to enhance the key sensitivity. The diffusion operation is implemented to resist the chosen plaintext attack. Through the analysis of the experimental results, we obtained the following conclusions:

1. The abundant Julia-like images are the copies of a Julia set and can be generated by a few parameters, which greatly reduces the key store space. The chaotic characteristic of the boundaries in the Julia image gives the key extreme sensitivity to the slight parameter changes, improving the security of the encryption algorithm greatly. In our experiments, the key sensitivity achieves 10^−15^.
2. The diffusion process has a good effect in the pixel spread, and provides much large key space, has a high sensitivity to the plain image and keys, and especially enhances the resistance against chosen plaintext attack.
3. The entropy value of the cipher image achieves an ideal value, illustrating that the encryption system not only has a good randomness but also can resist the entropy attack. The statistical analysis shows that the distributions of the cipher image are uniform, also indicating the success of the algorithm in randomizing the output. In addition, the randomness test passes the sp800-22 test suite, proving the randomness of the cipher image on another side.

For future work, we will consider choosing better Julia set keys and other methods to improve the algorithm for the key conversion.
